# Cardioembolic stroke in Takayasu arteritis treated with dual stent retriever via brachial access: A case report

**DOI:** 10.1016/j.radcr.2025.10.015

**Published:** 2025-11-15

**Authors:** Gamaliel Wibowo Soetanto, Elvan Wiyarta, Fritz Sumantri Usman

**Affiliations:** a Department of Neurology, St Borromeus Hospital, Bandung, 40132, Indonesia; b Intensive Care Unit, University of Indonesia Hospital, Depok, 16424, Indonesia; c Department of Neurology, Pelni Hospital, Jakarta, 11410, Indonesia

**Keywords:** Takayasu arteritis, Cardioembolic stroke, Stent, Brachial access, Mechanical thrombectomy, Case report

## Abstract

Takayasu arteritis (TA) is a large-vessel vasculitis that may predispose to ischemic stroke through arterial stenosis, dissection, in-situ thrombosis, or cardiac embolism. We report the case of a 36-year-old man with TA who presented with acute left hemiplegia due to right MCA M1 occlusion. Because of extensive aortoiliac disease, mechanical thrombectomy was performed via right brachial access using two stent retrievers across the M1 bifurcation, achieving single-pass complete reperfusion (mTICI 3). Post-procedural echocardiography revealed a large mobile apical left-ventricular thrombus, indicating a cardioembolic mechanism, and anticoagulation was initiated. The patient recovered to NIHSS 0 at discharge and mRS 1 at 3 months. This case highlights the feasibility of brachial access and dual stent retriever technique in TA patients with inaccessible femoral arteries, and underscores the importance of individualized access and device strategies in complex vascular anatomy.

## Introduction

Takayasu arteritis (TA) is a rare, chronic large-vessel vasculitis that predominantly affects young women and involves the aorta and its major branches. The estimated global incidence is approximately 1.11 cases per million per year, with higher prevalence reported in Asia, including Southeast Asia [1,2]. While neurological complications in TA are relatively uncommon, stroke has been reported as the presenting manifestation in about 6.3% of cases, often due to stenosis or occlusion of intracranial vessels such as the middle cerebral artery (MCA) [3]. Cardioembolic stroke due to inflammatory cardiomyopathy and ventricular thrombus formation in TA is even rarer, highlighting the systemic inflammatory burden associated with this disease.

Endovascular thrombectomy has become the standard of care for large vessel occlusion (LVO), but vascular access may be challenging in TA, especially when extensive peripheral arterial disease precludes femoral entry. Here, we report a case of a 36-year-old male with known Type V TA who presented with acute ischemic stroke due to a cardioembolic right M1 occlusion. While alternative access routes and dual stent retriever techniques have been reported in selected cases of challenging anatomy, to our knowledge this is the first published case describing successful dual stent retriever thrombectomy via brachial access in a patient with TA.

## Case illustration

A 36-year-old Indonesian male, previously independent in daily activities with no known cardiovascular risk factors, presented to the emergency department with acute-onset left hemiplegia and slurred speech, which began approximately 90 minutes prior to arrival. There was no history of hypertension, diabetes mellitus, smoking, or prior cerebrovascular events. He had no known family history of stroke or autoimmune disease and no significant psychosocial stressors.

**Fig. 1:**
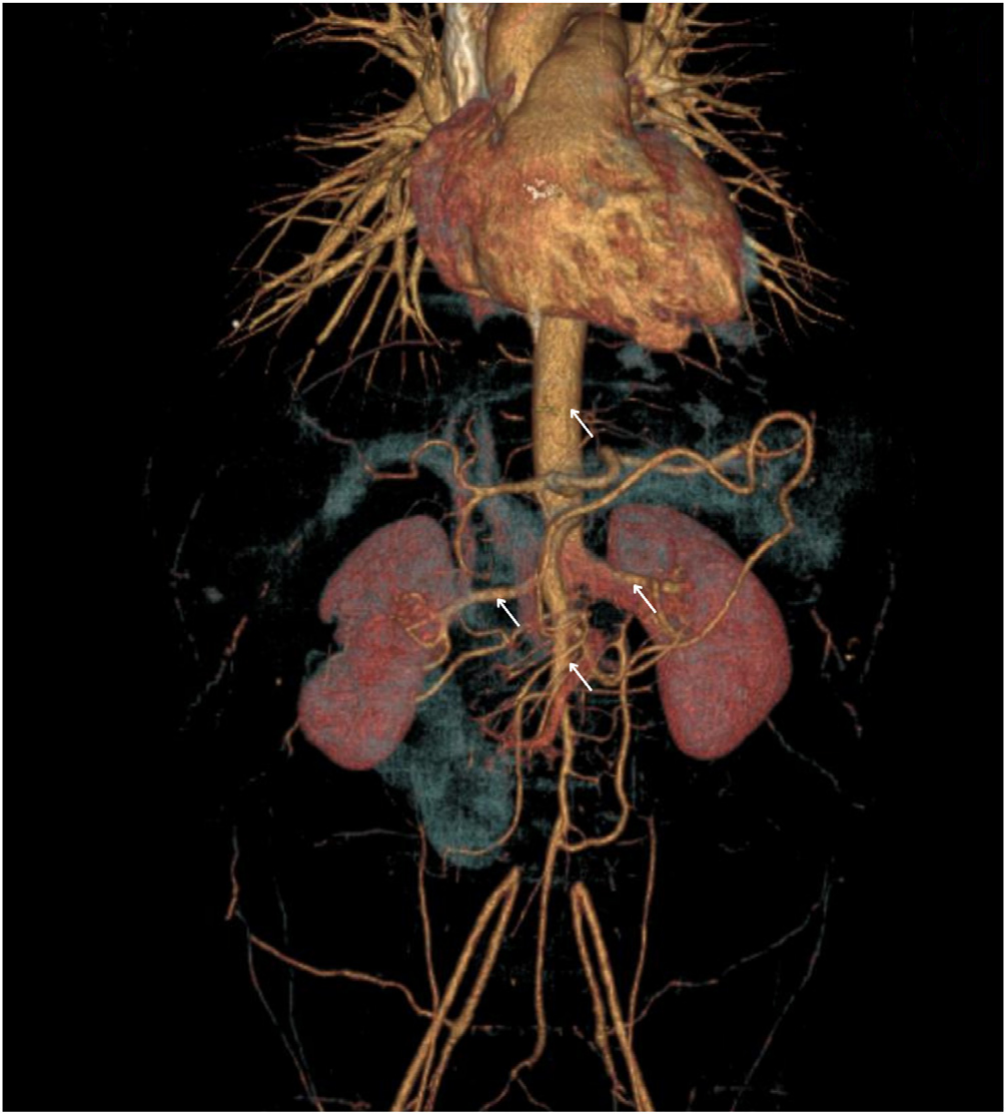
3D reconstruction of chest-abdomen CTA showing extensive large-vessel involvement (arrow) typical of Type V Takayasu arteritis.

Six months prior to presentation, the patient experienced progressive fatigue, bilateral lower limb claudication, and diminished lower extremity pulses. Physical examination at that time revealed weak femoral pulses, a systolic blood pressure discrepancy between arms, and a soft systolic murmur. Laboratory work-up showed markedly elevated erythrocyte sedimentation rate (ESR) and C-reactive protein (CRP) levels. A computed tomography (CT) angiography of the chest and abdomen revealed circumferential wall thickening and luminal narrowing of the thoracic and abdominal aorta, renal arteries, and bilateral iliac and femoral arteries (Fig. 1), confirming a diagnosis of Type V TA. The patient was subsequently initiated on subcutaneous tocilizumab therapy, with stable clinical response during follow-up. No antiplatelet or anticoagulant therapy was prescribed.

On arrival to the emergency department, physical examination revealed left hemiplegia, left facial droop, and dysarthria, yielding a National Institutes of Health Stroke Scale (NIHSS) score of 14. Vitals were stable. Cardiopulmonary auscultation was normal except for a soft systolic murmur. No carotid bruits were noted. Peripheral pulses were weak but symmetric, with markedly diminished bilateral femoral pulses.

Initial laboratory tests demonstrated elevated C-reactive protein (CRP) and erythrocyte sedimentation rate (ESR), with normal renal function and coagulation profile. Electrocardiography showed sinus rhythm. A non-contrast head CT, performed as part of the acute stroke protocol, revealed no hemorrhage or early ischemic changes. CT angiography (CTA) of the brain and neck confirmed a right M1 segment occlusion with poor collateral flow.

Given the patient’s young age, history of systemic vasculitis, and presence of LVO, a broad differential diagnosis was considered, including vasculitis-related in-situ thrombosis, intracranial dissection, hypercoagulable state, and cardioembolism. In the absence of preceding trauma or overt coagulopathy, and in the context of known large-vessel TA, an embolic mechanism from a cardiac or proximal aortic source was strongly suspected, though the exact origin remained unclear at this stage

**Fig. 2:**
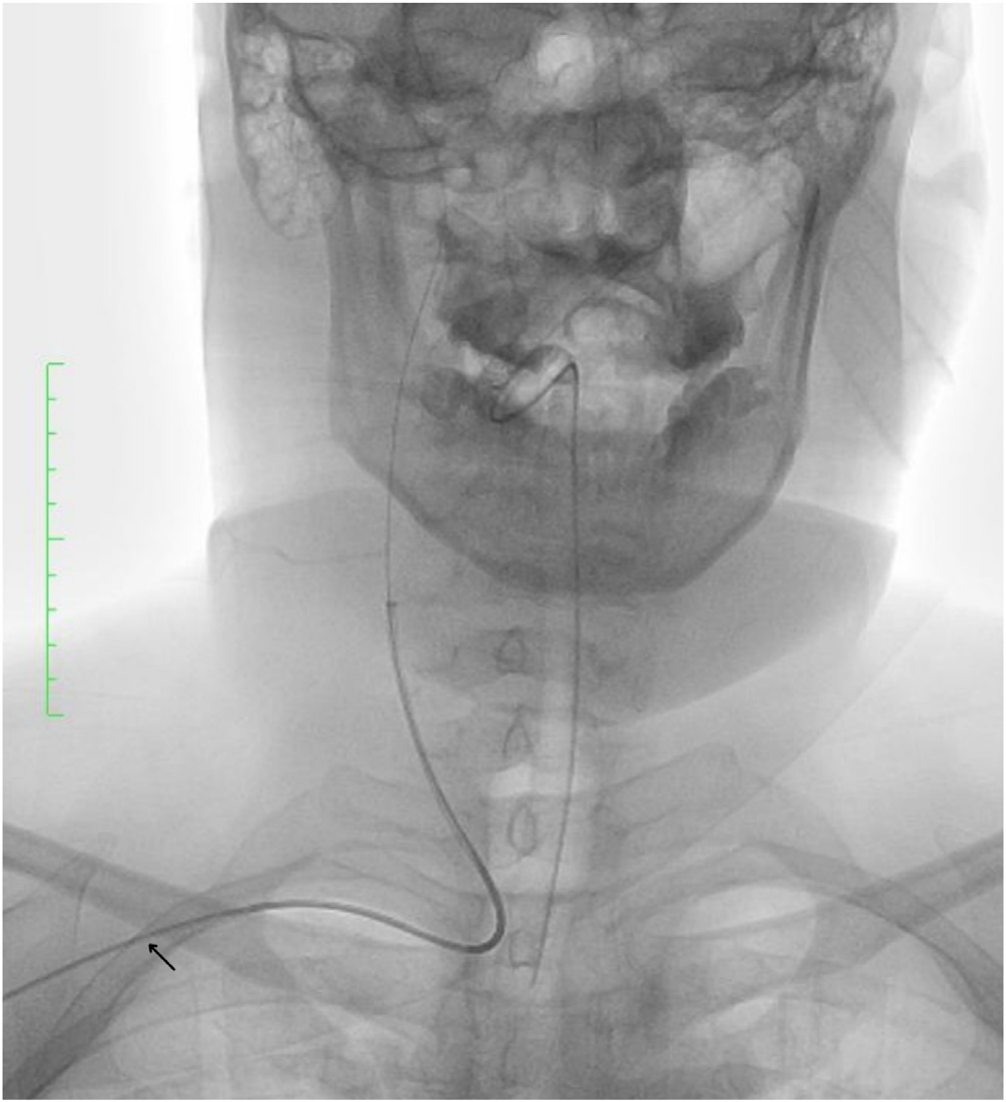
Fluoroscopy image demonstrating right brachial access (arrow).

Due to severe peripheral vascular disease, femoral access was deemed unfeasible. The patient underwent emergency endovascular thrombectomy via right brachial artery access (Fig. 2). Under local anesthesia and systemic heparinization, vascular access was established using a 6 Fr radial introducer sheath (Terumo Corporation). A Benchmark 6 Fr 071 guiding catheter (Penumbra Inc.) was advanced over a Hybrid 14 D 0.014 guidewire (Balt) and navigated under fluoroscopic guidance into the right internal carotid artery (ICA).

**Fig. 3:**
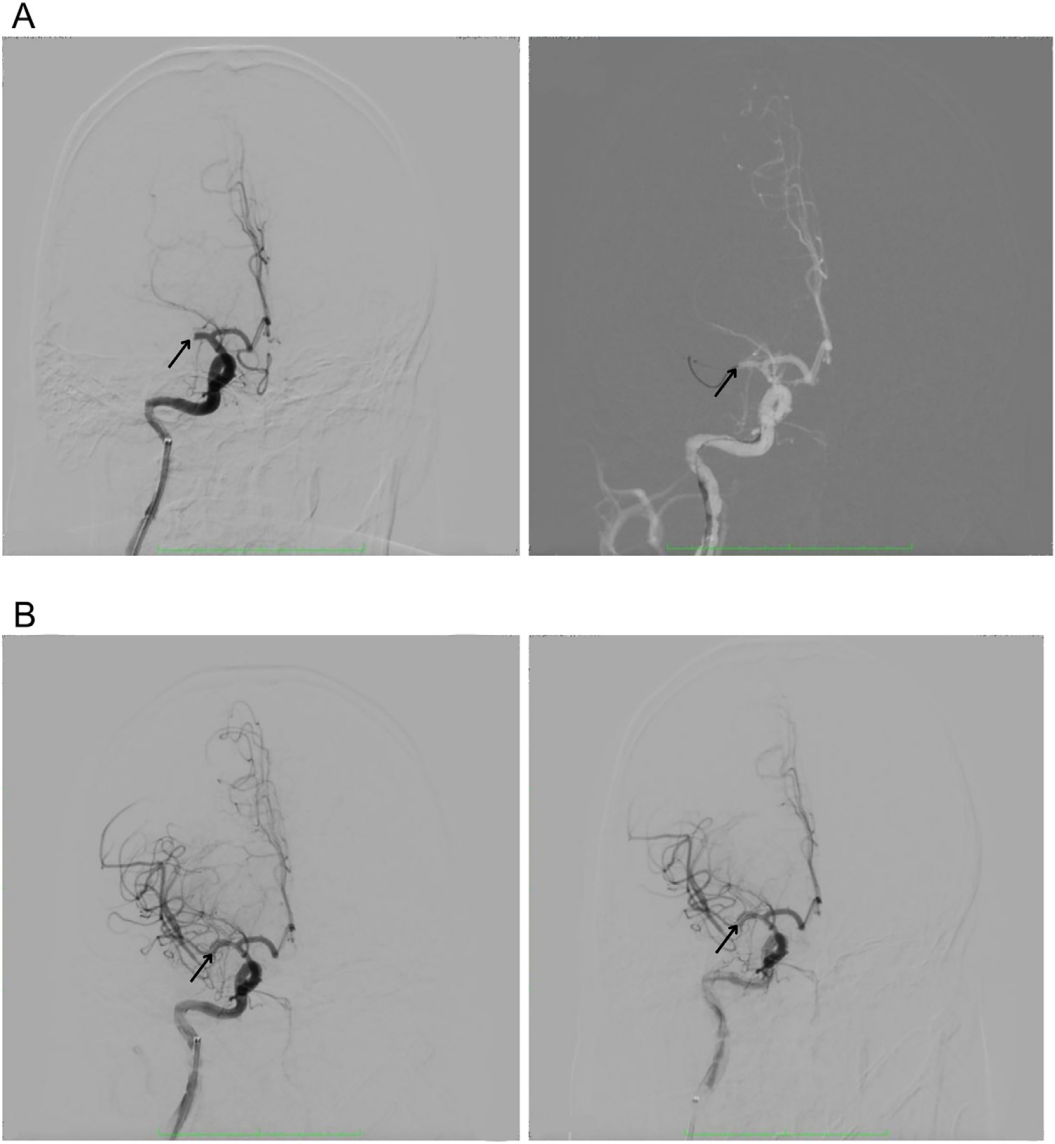
Digital subtraction angiography findings.(A) Pre-thrombectomy DSA demonstrating abrupt occlusion of the right M1 segment with absence of distal MCA branch filling (arrow), consistent with TICI 0. (B) Post-thrombectomy DSA showing complete reperfusion of the MCA territory with restoration of distal flow (arrow), consistent with eTICI 3.

Digital subtraction angiography (DSA) revealed a complete occlusion of the right M1 segment of the middle cerebral artery with no distal flow (TICI 0) (Fig. 3A). DSA was the sole imaging modality performed in this case; no CTA or MRI was obtained. Given the anticipated high clot burden and bifurcated clot location, a dual stent retriever strategy was planned to optimize first-pass success.

A Vasco 10 microcatheter (Balt, ID 0.017) was advanced into the superior M2 trunk, and a Vasco 18 microcatheter (Balt, ID 0.021) was positioned in the inferior M2 trunk. Through these catheters, a CatchView Mini 4 × 29 mm stent retriever and a CatchView Maxi 6 × 67 mm stent retriever (Balt) were deployed simultaneously, spanning the entire length of the occluded M1 segment and partially engaging the distal clot in the bifurcated M2 branches.

After a dwell time of 3 minutes to ensure stent-thrombus integration, both devices were retracted simultaneously ("in tandem") under continuous aspiration via the Benchmark catheter. Proximal flow control was maintained during retrieval using a balloon occlusion technique. Complete reperfusion (mTICI 3) was achieved in a single pass, as confirmed by follow-up DSA showing restored antegrade flow in all MCA branches (Fig. 3B). No procedure-related complications such as vasospasm, dissection, or distal emboli were observed.

Following the procedure, the patient was admitted to the stroke unit. On day 1, his NIHSS improved to 6, and by day 5, it further improved to 3, with substantial recovery of motor function and speech.

**Fig. 4:**
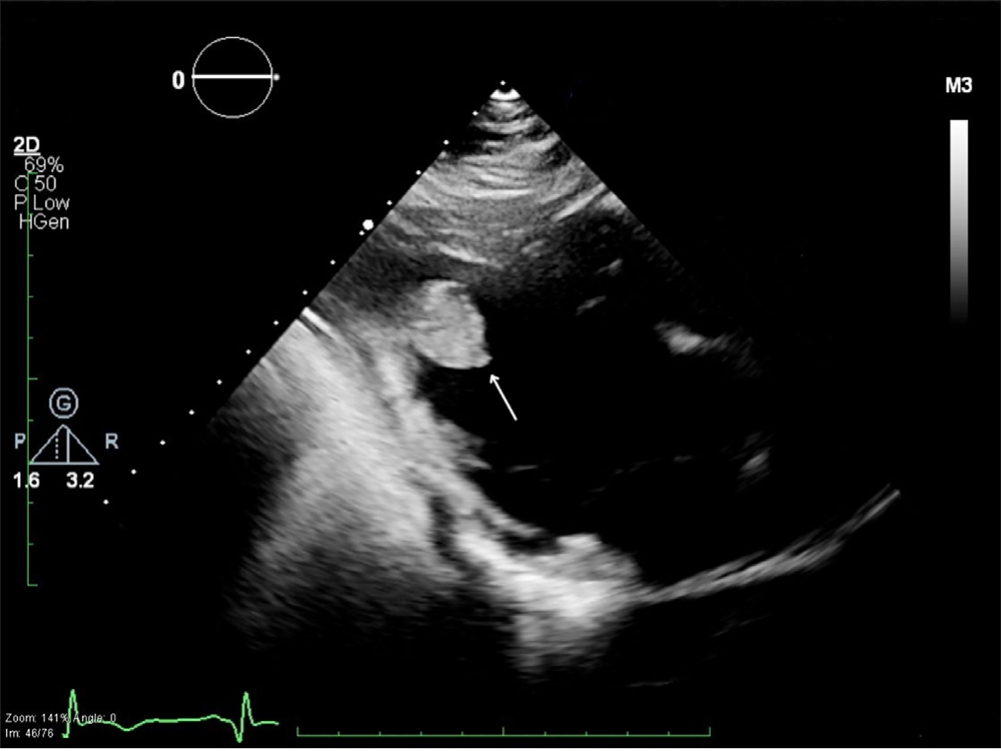
Echocardiographic report demonstrating LVEF 46%, regional wall motion abnormalities, and LV thrombus (arrow).

As part of in-hospital etiologic evaluation, a transthoracic echocardiogram (TTE) was performed and revealed a mobile thrombus measuring 2.0 × 4.1 cm at the apex of a dilated left ventricle, along with eccentric hypertrophy and a reduced ejection fraction (46%). Regional wall motion abnormalities, particularly in the anterior and inferoseptal segments, were consistent with Takayasu-related inflammatory cardiomyopathy. These findings confirmed the suspicion of a cardioembolic stroke, with the thrombus likely originating from the inflamed, hypokinetic ventricular segment (Fig. 4).

The patient was initiated on oral anticoagulation with warfarin, overlapping with dual antiplatelet therapy during transition. No bleeding or thromboembolic complications occurred during hospitalization.

At 3-month follow-up, the patient had achieved NIHSS 0 and modified Rankin Scale (mRS) 1, indicating near-complete neurological recovery. Follow-up CTA showed no new vascular lesions.

## Discussion

This case highlights a rare presentation of cardioembolic stroke in a young patient with TA, effectively managed using a dual stent retriever thrombectomy via brachial access. The coexistence of a mobile left ventricular thrombus, LVO, and inaccessible femoral arteries due to diffuse vasculopathy posed significant therapeutic challenges, necessitating an individualized endovascular strategy. The procedure achieved complete recanalization (mTICI 3) in a single pass, resulting in excellent neurological recovery and reinforcing the feasibility of complex thrombectomy in patients with altered vascular anatomy.

In this case, the diagnosis of Type V TA was critical in shaping both the clinical presentation and the interventional decision-making process. Type V disease, characterized by widespread involvement of both the aortic arch and abdominal aorta with their major branches, often leads to progressive luminal narrowing, impaired perfusion, and increased systemic thromboembolic risk [4]. The patient exhibited bilateral iliofemoral occlusion, which precluded transfemoral access, and required brachial artery access for neurointervention. Beyond structural stenosis, TA can also cause myocardial involvement, resulting in inflammatory cardiomyopathy, left ventricular dysfunction, and ventricular thrombus formation due to regional wall motion abnormalities and endothelial injury [5]. This pathophysiological link between vasculitis and cardioembolic stroke has been increasingly recognized, though rarely reported in procedural literature. Importantly, the systemic inflammatory state in TA may contribute to a prothrombotic milieu, further complicating both acute stroke management and postprocedural anticoagulation strategies.

Mechanical thrombectomy has become the standard treatment for acute LVO, with stent retrievers widely adopted as first-line tools [6]. Although transfemoral access is conventional, anatomical limitations such as severe peripheral artery disease or aortic arch tortuosity can make this route unfeasible. In such cases, brachial access may serve as a safe and effective alternative. Tsuji et al. reported a series demonstrating the feasibility of transbrachial thrombectomy in patients with difficult vascular anatomy, without significant increase in procedural complications [7].

Additionally, although not yet standard practice, the dual stent retriever technique has gained interest in selected cases of high clot burden or complex occlusion geometry, such as bifurcation clots. Vega et al. performed a systematic review evaluating double stent retriever use as a first-line strategy in M1 and terminal ICA occlusions and found high rates of successful recanalization (TICI ≥2b) and acceptable safety profile, particularly when dual deployment improved device-clot integration [8]. This “cross-compression” or “pincer” mechanism offers an advantage in thrombus entrapment and may increase the likelihood of first-pass effect, as observed in our patient.

This case emphasizes several key considerations for clinical practice. First, access route selection should be individualized, particularly in patients with vasculitis-related arterial disease. Second, advanced thrombectomy strategies such as dual device deployment should be considered in appropriately selected cases to optimize recanalization outcomes. Finally, this report adds to the limited but growing literature supporting the use of alternative access and advanced mechanical techniques in complex neurointerventional scenarios. Further prospective studies and registry data are needed to better define the role, indications, and safety profile of these approaches.

As a single case, generalizability is limited. Imaging was restricted to DSA and TTE, since we did not obtain transesophageal echocardiography or cardiac MRI. Extended rhythm monitoring and long-term cardiology follow-up were also unavailable, so alternative cardioaortic sources cannot be excluded. Despite this uncertainty, the clinical course supports a cardioembolic mechanism and illustrates the feasibility of brachial access with a dual stent retriever in challenging anatomy.

## Conclusion

This case illustrates the successful management of a young male patient with TA presenting with acute ischemic stroke due to a cardioembolic event. The utilization of a dual stent retriever thrombectomy via brachial access led to complete recanalization and favorable neurological recovery. This approach underscores the importance of individualized endovascular strategies in patients with complex vascular pathologies where traditional access routes are compromised.

## Ethics approval and consent to participate

The St Borromeus Hospital, Bandung Institutional Review Board, approved the report protocols. Informed consent for participation was obtained from all subjects involved in the study.

## Availability of data and materials

All data generated or analyzed during the study are included in this published article.

## Authors’ contributions

Conceptualization, **GWS, EW, FSU:** Methodology, **GWS, EW:** Software, **GWS, EW:** Validation, **GWS, EW, FSU:** Formal analysis, **GWS, EW:** Investigation, **GWS, EW, FSU:** Resources, **GWS, FSU:** Data curation, **EW:** Writing—original draft preparation, **GWS, EW:** Writing—review and editing, all authors; Visualization, **GWS, EW:** Supervision, **FSU:** Project administration, **EW:** Funding acquisition, **FSU**. All authors have read and agreed to the published version of the manuscript.

## Patient consent

Informed consent was obtained from all subjects involved in the study.

## References

[1] Rutter M., Bowley J., Lanyon P.C., Grainge M.J., Pearce FA. (2021). A systematic review and meta-analysis of the incidence rate of Takayasu arteritis. Rheumatology (Oxford).

[2] Sun Y., Yin M.M., Ma L.L., Dai X.M., Lv L.J., Chen X.X. (2021). Epidemiology of Takayasu arteritis in Shanghai: a hospital-based study and systematic review. Int J Rheum Dis.

[3] Zhang G., Ni J., Yang Y., Li J., Tian X., Zeng X. (2023). Clinical and vascular features of stroke in Takayasu's arteritis: a 24-year retrospective study. Rheumatol Immunol Res.

[4] Saadoun D., Vautier M., Cacoub P. (2021). Medium- and large-vessel vasculitis. Circulation.

[5] Álvarez Vázquez A., López Alcolea J., Urmeneta Ulloa J., Forteza Gil A., Rivas Oyarzabal J., Cabrera Rodríguez J. (2025). Takayasu's arteritis causing coronary stenosis with myocardial ischemia, severe aortic regurgitation, and pericarditis. Radiol Case Rep..

[6] Parvathy G., Dey R.C., Kutikuppala L.V.S., Maheshwari A.R., Josey E., Chintala J.S. (2023). Mechanical thrombectomy for AIS from large vessel occlusion - current trends and future perspectives. Ann Med Surg.

[7] Tsuji Y., Miki T., Kakita H., Sato K., Yoshida T., Shimizu F. (2020). Mechanical thrombectomy for large vessel occlusion via the transbrachial approach: case Series. Neurointervention.

[8] Vega P., Murias E., Jimenez J.M., Chaviano J., Rodriguez J., Calleja S. (2022). First-line double stentriever thrombectomy for M1/TICA occlusions: initial experiences. Clin Neuroradiol.

